# Sepsis in Frail Older Adults: Tailored Antimicrobial Stewardship and Individualized Care Approach

**DOI:** 10.3390/antibiotics15050496

**Published:** 2026-05-14

**Authors:** Elisa Fabbri, Gianpiero Tebano, Arianna de Angelis, Annaviola Del Prete, Lorenzo Maestri, Francesco Cristini, Paolo Muratori

**Affiliations:** 1 Department of Medical and Surgical Sciences, University of Bologna, 40126 Bologna, Italy; 2 Division of Internal Medicine, Morgagni-Pierantoni Hospital, 47121 Forlì, Italy; 3 Infectious Disease Unit, Morgagni-Pieratoni Hospital, 47121 Forlì, Italy; 4 Department for Life Quality Studies, University of Bologna, 40126 Bologna, Italy

**Keywords:** sepsis, older adults, frailty, antibiotics, antimicrobial stewardship

## Abstract

Frail older adults face an increased risk and severity of sepsis, which contributes to a notably high mortality rate. The management of sepsis in this population presents significant challenges, such as diagnostic complexity, a higher prevalence of multidrug-resistant pathogens, difficulties in achieving effective source control, and an increased risk of adverse events and toxicity associated with antibiotic therapy. In addition, accurate prognostic evaluation based on a comprehensive geriatric assessment is essential to determine the intensity of care required and to develop a personalized plan of care. Despite these considerations, frail older adults are still often underrepresented in randomized clinical trials and guidelines. In this narrative review, we discuss the main pillars of tailored antimicrobial stewardship in frail older adults. We propose a practical, stepwise approach to individualized care, delivered by a multidisciplinary team and based on a careful balance between treatment intensity and patients’ vulnerabilities, needs, and priorities.

## 1. Introduction

Sepsis is a life-threatening organ dysfunction resulting from a dysregulated response of the host to infection [1,2,3,4]. Despite medical advances and a better understanding of its pathophysiology, sepsis remains one of the leading causes of death globally. It has been estimated to account for approximately 20% of global deaths, based on Global Burden of Disease analyses [5,6].

This can be attributed in part to the aging population and the changing demographics of sepsis, which are shifting towards older age groups. In fact, the occurrence of sepsis is markedly higher among older adults and the prognosis for these patients is considerably poorer [7,8,9,10]. Individuals aged 65 or older have been reported to have up to a 13-fold higher incidence of sepsis compared with younger adults in a large longitudinal observational study using US hospital discharge data [11]. Furthermore, the estimated mortality rate for older patients admitted to the hospital with sepsis has been reported to range between 30% and 60%, depending on clinical severity and care setting, with even higher estimates of 40% to 80% for those over 80 years old [12,13].

However, among older individuals, even those of the same chronological age, there is notable biological variability in risk profiles and prognosis. Frailty, a clinical geriatric syndrome characterized by reduced physiological reserve and increased susceptibility to stressors [14], captures this variability and represents a strong and independent indicator of mortality and adverse outcomes in older patients with sepsis [15,16,17,18,19,20,21,22,23,24,25,26,27,28,29].

Approximately 50–60% of older individuals admitted to the emergency department with suspected sepsis are frail [25]. In addition, sepsis frequently complicates non-infectious conditions in frail older patients [10].

Despite this, frail older patients are significantly underrepresented in randomized clinical trials (RCTs) [30]. Most available RCTs on sepsis include younger and less complex populations [31], limiting external validity, while evidence specifically addressing frail older patients is largely derived from observational studies. Indeed, such studies are subject to confounding, selection bias, and heterogeneity in frailty definitions, which may affect the robustness and comparability of findings. As a result, clinical decision-making often relies on extrapolation from non-frail populations, underscoring the need for individualized approaches. Future research should prioritize the inclusion of frail older adults and consider pragmatic trial designs or prospective cohort studies with frailty-stratified analyses to enhance the applicability of evidence in this population [32].

Importantly, optimizing the management of frail older adults with sepsis requires a tailored, patient-centered approach that incorporates accurate prognostic assessment [10].

In this narrative review, we aim to summarize the specific characteristics of frail older patients with sepsis and to provide a comprehensive overview of the main evidence supporting antimicrobial stewardship and personalized management strategies for sepsis in this population.

## 2. Methods

This narrative review summarizes the current evidence on the management of sepsis in frail older adults, with a particular focus on antimicrobial stewardship and individualized care. A literature search was conducted using PubMed (Bethesda, MD, USA), using combinations of the following keywords: “sepsis”, “frailty”, “older adults”, “frail older adults”, “antibiotics”, “antimicrobial stewardship”, “de-escalation”, and “multidrug-resistant organisms”. Randomized controlled trials (RCTs), observational studies, meta-analyses, systematic reviews, and relevant clinical guidelines published in the past decade were considered. Earlier studies were also included when they provided important background or contributed to key concepts. Priority was given to studies focusing on older or frail patients with sepsis or severe infections. However, given the limited representation of frail individuals in clinical trials, we also included studies conducted in broader adult populations when their findings were considered clinically relevant. More than 500 records were initially identified and screened. Articles not published in English were excluded. Titles and abstracts were reviewed by the authors, and the reference lists of selected papers were examined to identify additional relevant studies. Full texts were retrieved for articles considered potentially eligible. The selection process was based on qualitative assessment of methodological robustness and relevance to the aims of the review, with particular attention to frailty, antimicrobial stewardship, and personalized management strategies. In total, 149 references were included to support this review. During the preparation of this manuscript, the authors used Grammarly, V1.2.259.1886 (Grammarly Inc. (San Francisco, CA, USA)) exclusively for English editing.

## 3. Characteristics of Frail Older Patients

Frailty is a prevalent clinical condition among older adults, characterized by diminished physiological reserves and a reduced capability to cope with stress, which increases the likelihood of negative outcomes [14,33]. The aging process is characterized by a gradual decline in the effectiveness of both cell-mediated and humoral immune responses (immunosenescence), leading to a greater vulnerability to infections and more severe manifestations of those infections [34]. This deterioration in immune function arises from biological mechanisms inherent to the aging process, including a persistent state of low-grade chronic inflammation referred to as inflammaging [35,36,37,38]. Chronic inflammation accelerates aging and contributes to the development of frailty, which is closely linked to immunosenescence and an elevated susceptibility to infections [39]. Moreover, in frail older adults, the emergence of an infection serves as an acute stressor for an organism that is already significantly compromised in its functional reserves, triggering a series of events that are often irreversible and potentially fatal [33].

Multimorbidity, malnutrition, sarcopenia, and other geriatric syndromes frequently occur in frail older individuals, contributing to an increased risk of severe infections [10]. Polypharmacy also represents a common feature in frail older adults and plays a relevant role, mainly due to the possibility of using medications that could lead to or exacerbate immunosuppression, along with a heightened risk of adverse drug reactions and drug-to-drug interactions (ADRs).

Notably, different pathophysiological changes can affect the pharmacokinetics and pharmacodynamics of antibiotics in older individuals [40,41,42,43,44,45,46]. For example, delayed gastric emptying, reduced splanchnic blood flow, and alterations in gastric pH can affect the bioavailability of orally administered medications [43]. Changes in body composition due to aging can impact how drugs are distributed, a situation that becomes even more pronounced in frail older persons where malnutrition and sarcopenia are usually present. Specifically, a reduction in adipose tissue leads to lower amounts of lipophilic drugs being stored, while changes in muscle mass and body fluid distribution affect how hydrophilic drugs are distributed. Notably, the reduction in total body water and the common occurrence of dehydration among older and frail adults are associated with a decreased volume of distribution of hydrophilic antibiotics, such as beta-lactam antibiotics, which results in higher plasma levels, and an increased risk of toxicity [46]. Moreover, in frail older individuals, renal clearance and hepatic metabolism are frequently impaired, which increases the risk of drug toxicity and adverse events. For example, reduced kidney function accounts for elevated vancomycin levels in older adults [47]. Therefore, at equivalent doses, vancomycin produces higher plasma concentrations in older individuals than in younger ones, which is associated with an increased risk of nephrotoxicity [48].

Approximately 16% of hospitalized older patients experience significant adverse drug reactions, with antibiotics responsible for nearly 15% of these cases [49]. Gastrointestinal side effects are common in frail older adults, including antibiotic-induced diarrhea and gut microbiome disruption, which promotes the growth of opportunistic pathogens like Clostridioides difficile [50]. Other relevant examples include higher rates of carbapenem-related seizures [51] and an increased risk of arrhythmia associated with azithromycin and levofloxacin [52].

Furthermore, it is important to highlight that frail older patients often reside in long-term care facilities, experience repeated hospital admissions, have indwelling devices, and are frequently exposed to antibiotics. These characteristics increase the risk of developing severe and complicated infections caused by multidrug-resistant organisms (MDROs), including extended-spectrum β-lactamase (ESBL)-producing Escherichia coli, ESBL-producing Klebsiella pneumoniae, carbapenem-resistant (CR) Enterobacterales, methicillin-resistant Staphylococcus aureus (MRSA), vancomycin-resistant Enterococcus spp. (VRE), multidrug-resistant (MDR) Pseudomonas aeruginosa and Acinetobacter baumannii [53,54]. The two fundamental pillars in the fight against antibiotic resistance are the containment of MDRO transmission through infection-control measures and the promotion of responsible antibiotic use. Therefore, antimicrobial stewardship, which consists of various strategies to promote the responsible use of antibiotics [55], is crucial in frail older adults in order to mitigate the negative impact of antibiotic resistance on patient outcomes [56].

**Figure 1:**
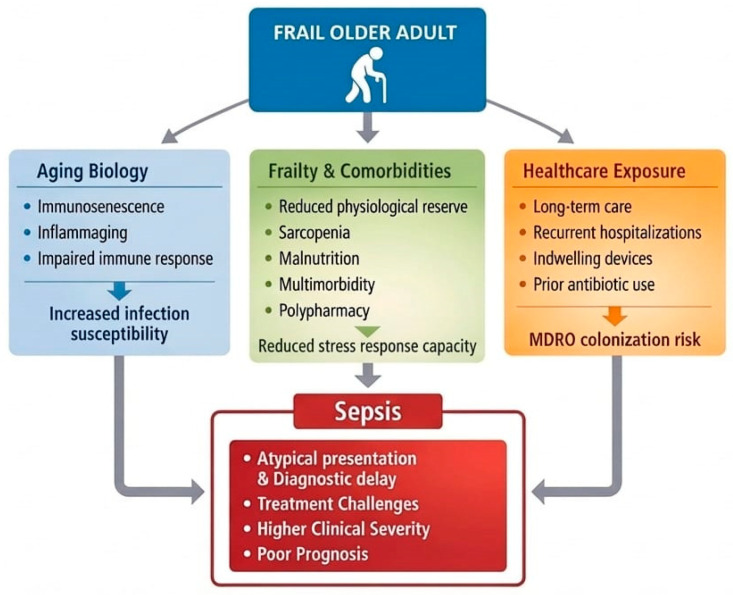
Determinants of increased sepsis vulnerability in frail older adults.

Figure 1 shows the combination of characteristics that makes frail older individuals especially susceptible to sepsis, leading to heightened clinical severity, higher likelihood of complications, and, as discussed in the subsequent paragraphs, greater challenges in diagnosis and treatment that culminate in a poorer prognosis.

## 4. Antimicrobial Stewardship in Frail Older Adults with Sepsis

### 4.1. Early Recognition of Sepsis and Diagnostic Accuracy

Sepsis is a time-dependent disease, and the main determinant of prognosis is the prompt identification of the condition and the timely initiation of appropriate empiric antibiotic treatment. According to the Surviving Sepsis Campaign guidelines, empiric antibiotic therapy should be started as soon as possible, specifically within one hour in cases of septic shock and within three hours in cases of sepsis [57]. A significant challenge in diagnosing sepsis among frail older adults is its atypical presentation, which can lead to delays in starting antibiotic treatment [58] and a consequent increased risk of death [59]. Fever may often be absent [60], while nonspecific symptoms such as confusion, delirium, fatigue, and decline in functional ability are more frequent [61]. For both adults and older individuals, various scoring systems—Sequential Organ Failure Assessment (SOFA), quick SOFA (qSOFA), National Early Warning Score (NEWS), and NEWS2—have been proposed to evaluate organ damage and aid in the early detection of sepsis. The 2026 Surviving Sepsis Campaign guidelines recommend NEWS over qSOFA as a single screening tool for sepsis because of its greater sensitivity [57]. Consistently, a previous study has shown that qSOFA alone does not adequately capture the severity of sepsis in older adults [62]. Despite these findings, there remains a persistent lack of scoring systems specifically designed for older or frail patients, a gap not addressed by current guidelines. Additionally, as summarized in Table 1, studies evaluating the performance of existing scoring systems for screening or prognostic assessment in older patients have reported conflicting results [24,62,63,64,65,66,67,68,69,70,71,72,73]. These studies are characterized by considerable heterogeneity in settings, ranging from the emergency department (ED) to the intensive care unit (ICU). Moreover, they are exclusively observational, with most being retrospective and therefore carrying a risk of selection bias, as they often include only patients with suspected infection. One study comparing these scores for identifying sepsis in older patients with suspected infection found that NEWS did not perform substantially better than qSOFA in the geriatric population [71]. This study further indicated that, while qSOFA may be useful for ruling out sepsis in patients with suspected infection, it lacks utility in supporting the diagnosis because of its low specificity. In response to this limitation, other authors have emphasized the need to combine qSOFA with frailty scales to enhance its predictive effectiveness [15,64,74]. For instance, a geriatric-qSOFA, which substitutes delirium evaluation for level of consciousness assessment, demonstrated a significant association with short-term mortality and provided improved predictive capability in older patients compared to the conventional qSOFA [68]. This study is limited by its small retrospective design and the absence of externally validated, peer-reviewed outcomes. Larger prospective studies are required to develop and validate specific scoring systems for sepsis identification and prognostic assessment in older patients with infection. Subsequently, randomized controlled trials may be necessary to determine whether implementing these tools improves clinical decision-making and patient outcomes.

Additional support for the diagnosis is provided by biomarkers. C-reactive protein (CRP) is a rapid, economical, and widely available biomarker that is frequently used in geriatric patients [75]. However, its levels may be non-specifically elevated due to a range of underlying chronic pro-inflammatory conditions. Notably, a prior study developed a clinical prediction model that integrates clinical characteristics (such as systolic blood pressure and oxygen saturation) with CRP, demonstrating strong discriminative ability for identifying serious infections in older patients in the emergency department [76]. In this case as well, external validation is required to confirm the generalizability of this model.

The diagnostic performance of procalcitonin (PCT) for bacteremia and sepsis in older patients does not appear to be inferior to that of younger individuals [77], although higher cut-off thresholds have been proposed, especially for older adults with chronic kidney disease. A prior study confirmed the diagnostic accuracy of PCT in frail older patients for diagnosing sepsis, identifying 1.4 ng/mL as the optimal threshold in this population [78]. Nevertheless, the small sample size of this study (140 participants) may restrict the applicability of these findings, and larger studies are needed to confirm them.

### 4.2. Avoiding Unnecessary Antibiotic Treatment

Before starting empirical treatment, it is fundamental to have a clear rationale for antibiotic use and to discontinue therapy when it is not needed. In viral infections such as influenza or COVID-19, antibiotics are appropriate only when bacterial co-infection is suspected [79]. However, most hospitalized patients with influenza receive antibiotics, including over 30% without evidence of bacterial infection [79]. This overuse is even greater in COVID-19, where antibiotic prescribing reaches 75%, despite an estimated bacterial co-infection rate of 8.6% [80]. The likelihood of antibiotic prescription increases with patient age, and older outpatients and emergency department patients had particularly high rates of use during the COVID-19 pandemic [81]. Inappropriate antibiotic use contributes to increased resistance, especially among Gram-negative bacteria [82], without improving patient outcomes [83,84]. The implementation of rapid diagnostic techniques, such as multiplex PCR, facilitates the early detection of viral infections and helps prevent unnecessary antibiotic use [84]. Conducting prospective antimicrobial stewardship (AMS) audits and providing feedback has been shown to be effective in optimizing antibiotic therapy and reducing inappropriate prescriptions in patients admitted to the hospital with COVID-19 [85].

Another example of inappropriate antimicrobial prescribing involves low-risk catheter-associated bloodstream infections caused by coagulase-negative staphylococci, which can be effectively managed with catheter removal alone, without the need for antibiotic therapy, while maintaining efficacy and safety [86,87,88].

Moreover, frail older adults frequently receive antibiotics inappropriately due to the presence of asymptomatic bacteriuria [89,90]. Notably, a previous large European RCT demonstrated that a multifaceted antibiotic stewardship intervention safely reduced antibiotic prescribing for suspected urinary tract infections in frail older adults [89].

The presence of bacteria in urinary catheters or surgical drains in frail older adults typically indicates asymptomatic colonization rather than true infection [91,92]. Prescribing antibiotics in these situations is not appropriate, as these drugs do not reduce the risk of future urosepsis or systemic complications. Instead, they significantly increase the risk of negative outcomes, such as Clostridioides difficile infection (CDI) and the emergence of MDROs. Clinical guidelines emphasize that, in the absence of systemic symptoms like fever or sudden changes in mental status, the focus should be on removing or replacing the device rather than initiating antimicrobial treatment.

Finally, non-infectious systemic inflammatory response syndrome (SIRS) is highly prevalent in frail older adults due to surgery, trauma, or underlying chronic conditions. However, it is frequently mismanaged with inappropriate antibiotic therapy [93]. In the absence of a confirmed bacterial source, initiating antimicrobials for sterile inflammation offers no clinical benefit and contributes to gut microbiome disruption and the emergence of MDROs.

### 4.3. Source Control

Source control is crucial in sepsis management and includes draining abscesses, debridement of infected or necrotic tissues, and removal of infected devices [94]. Common indications for source control include abdominal abscesses, cholecystitis or cholangitis, orthopedic device infections, implantable device infections, and vascular catheter infections. Delayed or inadequate treatment of the infection source is associated with a substantial increase in mortality [94,95,96]. In frail older adults, source control procedures are frequently withheld because the perceived risks outweigh the potential benefits. However, evidence regarding the outcomes of source control in this population remains limited. Recent research suggests that source control interventions, even in patients aged 80 years and older, may improve survival rates [97].

### 4.4. Tailored Antimicrobial Therapy

When prescribing empiric antibiotic treatment for a frail older adult, it is necessary to consider factors related to the infection, the patient’s characteristics, and the local patterns of antibiotic resistance. The first point is to assess the likely source of infection, which, in older patients, most commonly involves the urinary and respiratory systems [98]. Additionally, it is important to consider the possibility of MDRO infection, taking into account both the patient’s individual risk factors and local epidemiological information [99]. Factors such as residence in a long-term care facility [53,100,101,102], a history of frequent hospital stays, recent antibiotic use [54], the presence of medical devices, underlying comorbidities, or immunosuppressive therapies [103,104] significantly increase the risk of developing antibiotic resistance. A previous study created a model that assigns points for different factors: age over 70 years (1 point), living in a long-term care facility (3 points), a history of cerebrovascular events (2 points), hospitalization within the past month (2 points), and recent use of antibiotics (2 points). The model’s optimal threshold for predicting colonization by multidrug-resistant Gram-negative bacteria and improving antibiotic therapy was determined to be ≥4 points [105]. Moreover, existing microbiological information, such as previous pathogen isolates, known colonization, or newly identified colonization status via surveillance swabs, should be incorporated into clinical decision-making, particularly in patients with sepsis [98,106].

### 4.5. Dosing

We have previously discussed how the aging process and the presence of comorbid conditions, such as renal impairment, along with geriatric syndromes like sarcopenia, malnutrition, and dehydration, influence the pharmacokinetics and pharmacodynamics of antibiotics. Therapeutic drug monitoring (TDM) is a clinical method used to assess medication levels to optimize individualized dosing regimens, particularly for aminoglycosides, glycopeptides, fluoroquinolones, beta-lactams, and linezolid [44]. Several studies have shown that TDM-guided dosing is beneficial in reducing drug-related adverse events, emergence of antibiotic-resistant strains, and clinical outcomes [107,108]. There are limited studies, predominantly retrospective and observational, that directly examine the role of TDM in optimizing antibiotic dosing for older individuals [44]. Nevertheless, implementing a TDM-based strategy may be particularly valuable in this vulnerable population, which faces a high prevalence of MDRO infections as well as an increased risk of drug-related toxicities [43].

### 4.6. De-Escalation

A pillar of antimicrobial stewardship is de-escalation, which involves transitioning from a broad-spectrum antibiotic treatment to a more targeted one, based on the findings from antibiotic susceptibility tests [109,110,111]. This approach is strongly advocated by global guidelines, and should ideally be implemented within 96 h from the start of antibiotic therapy [58]. Advantages include a reduction in antibiotic resistance, a lower likelihood of adverse effects, decreased risk of super infections such as CDI, shorter hospital stays, and lowered healthcare expenses [112,113,114]. Despite the limited number of randomized controlled trials (RCTs) [113], the existing literature substantially supports the safety of de-escalation in sepsis [110,115,116,117,118,119] and the Surviving Sepsis Campaign guidelines recommend a daily assessment for de-escalation rather than a fixed-duration treatment approach [58]. However, research specifically aimed at frail older individuals is lacking [30]. Notably, a recent study, including more than 36,000 hospitalized patients with community-onset sepsis, with a median age of 71 years, found that de-escalation on day 4 was associated with no increase in 90-day mortality, along with significantly shorter hospital stays and fewer days of total antibiotic exposure [119].

### 4.7. Duration of Antimicrobial Therapy

Determining the appropriate duration of antibiotic treatment for septic patients, particularly those who are older and frail, involves carefully balancing complete infection eradication with the avoidance of complications such as drug toxicity and resistance development. Recent studies suggest that for most sepsis cases with sufficient source control, shorter treatment durations are equally effective but safer than longer ones. This strategy decreases the likelihood of CDI and helps curb the rise of MDR strains. The recently published BALANCE trial, which included about 3600 patients with bacteremia, with a median age of 70 years, had a considerable representation of older and more vulnerable individuals, and found that a reduced length of antibiotic treatment (7 days compared to 14 days) was not inferior to longer treatment [120]. Moreover, evidence supports the use of PCT as a reliable and safe biomarker for stopping antibiotics in sepsis, particularly when it is part of a serial monitoring protocol and combined with the clinical evaluation of the patient [106,121]. In particular, the use of procalcitonin to guide initiation and duration of antibiotic treatment results in lower risks of mortality, lower antibiotic consumption, and lower risk of antibiotic-related side effects [122]. The efficacy of biomarkers, most notably PCT, to optimize the duration of antibiotic therapy has also been confirmed in older patients, with the advantage of avoiding both undertreatment and overtreatment [123,124].

### 4.8. Switch to Oral Therapy

While direct evidence remains limited for frail older adults, recent studies suggest that clinically stable, afebrile patients with well-controlled infection sources can safely transition to oral antibiotic therapy after a short course of intravenous antibiotics. For instance, this approach has been demonstrated to be beneficial in cases of uncomplicated Staphylococcus aureus bacteremia among low-risk individuals [125]. Moreover, based on findings from the POET study [126], in which the mean age was 67 years, the 2023 European Society of Cardiology (ESC) guidelines recommend that infective endocarditis caused by Enterococcus faecalis, Staphylococcus aureus, and coagulase-negative Staphylococci in clinically stable individuals may be transitioned to oral therapy after at least 10 days of intravenous treatment [127]. Similarly, evidence supports an early switch to oral therapy also in bone and joint infections [128]. Consistently, transition to oral antibiotic treatment has been shown to be safe for hospitalized patients with Gram-negative urinary tract or bloodstream infections [129,130,131]. This approach offers the benefit of decreasing the duration of hospital stays and the related complications, such as prolonged bed rest, delirium, and the development of antibiotic resistance, that are especially critical in frail older adults. This approach needs to be further investigated in critically ill septic patients (oral switch after clinical stability) and in older adults. Nonetheless, it is supported by substantial evidence in other settings and by a robust biological rationale.

## 5. Prognostic Evaluation, Level of Care Intensity and Ethical Aspects

Chronological age alone does not determine the intensity of care provided to older patients with sepsis; rather, it depends on an accurate evaluation of the patient’s prognosis through a comprehensive geriatric assessment, which includes pre-hospitalization functional status, comorbid conditions, cognition, nutritional status, and sarcopenia.

Table 2 summarizes previous studies investigating the impact of frailty on clinical outcomes, particularly in older patients hospitalized with sepsis [15,16,17,18,19,20,21,22,23,24,25,26,27,28,29]. Most studies show that frailty is a strong predictor of mortality and adverse outcomes. Notably, a recent study found that integrating frailty indices and the MDR status of culture isolates with conventional prognostic factors enhances the prediction of mortality in older patients with sepsis [24]. Furthermore, a Frailty Index based on laboratory test data (FI-Lab) has been shown to be an excellent predictor of mortality in older patients with sepsis [26] or with multidrug-resistant Klebsiella pneumoniae bloodstream infections [27]. However, a meta-analysis including thirty studies found that sarcopenia is strongly associated with in-hospital mortality and longer length of stay, while frailty is a less consistent predictor of mortality [28]. This finding likely does not reflect a lower clinical relevance, but rather greater methodological heterogeneity, driven by the lack of standardization in the definition and thresholds of frailty across studies.

**Figure 2:**
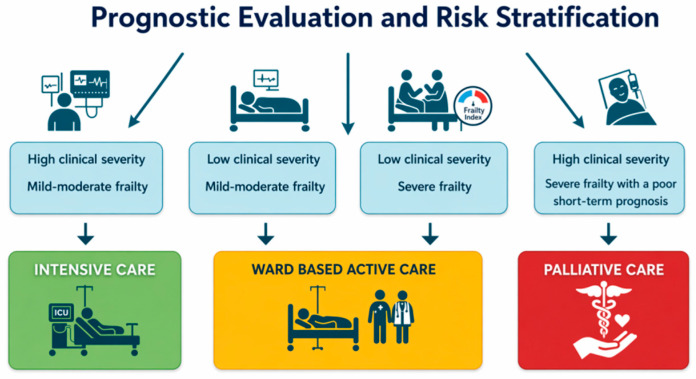
Prognostic evaluation and risk stratification for older patients with sepsis, based on clinical severity and frailty severity.

A personalized approach is essential when managing sepsis in older patients, particularly regarding the intensity of care. The treatment goal should be clearly defined, and admission to the intensive care unit (ICU) should be based on the anticipated clinical benefit. For patients with severe frailty, invasive interventions such as mechanical ventilation or dialysis are often disproportionate and unlikely to improve prognosis. Accurate prognostic stratification is therefore critical, considering not only the severity of the clinical presentation and microbiological risk, including multidrug-resistant (MDR) infections, but also the patient’s level of frailty. Frailty can be objectively assessed using validated tools such as the Frailty Index [132,133] or the Clinical Frailty Scale (CFS) [134]. Three levels of care can be identified for older patients with sepsis: (1) intensive care, for patients with high clinical severity and mild-to-moderate frailty; (2) ward-based active care, which consists of active management and treatment in internal medicine or acute geriatric wards without indication for escalation to intensive care, for patients with severe frailty; and (3) palliative care, for patients with high clinical severity and severe frailty and a poor short-term prognosis (Figure 2).

In this context, medical futility at the end of life refers to any intervention that offers no reasonable prospect of benefit, either in terms of life expectancy or quality of life [135]. The use of antibiotic treatment at the end of life remains a matter of ongoing debate [136,137], as it does not improve survival and is associated with increased risks of adverse events, including toxicity, drug interactions, CDI, and multidrug-resistant infections. Additionally, antibiotic administration may cause discomfort and inconvenience for the patient, particularly when intravenous infusions are required. The primary role of antibiotic therapy is curative, intended for infection eradication in patients with sufficient functional reserve and life expectancy. In terminally ill patients, antibiotics may be considered for palliative purposes, focusing solely on symptom control. When neither curative nor palliative indications are present, withholding or discontinuing antibiotics at the end of life is a reasonable approach, as long as it is shared with the patient and their family [138].

## 6. Conclusions and Future Directions

Managing sepsis in frail older patients poses one of the most intricate challenges in contemporary medicine. It requires prompt treatment, along with a careful balance between treatment intensity and patient vulnerabilities. These vulnerabilities arise from reduced functional capacity, diminished physiological reserves, and the coexistence of multiple comorbidities and geriatric conditions [56,139,140]. Precision medicine addresses this challenge by focusing on personalized antimicrobial treatment guided by tailored antimicrobial stewardship [141], and fostering collaboration among multidisciplinary teams [56,142,143], including geriatricians, internists, infectious disease specialists, pharmacologists, and intensivists.

**Figure 3:**
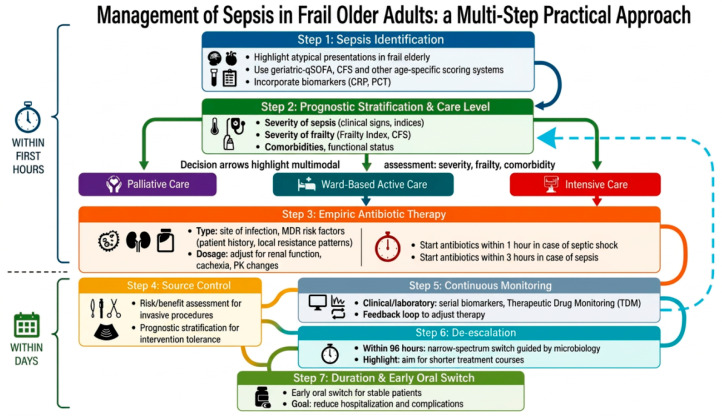
A multi-step practical approach for the management of sepsis in frail older adults. The dashed line indicates that, even during the monitoring phase, it is important to reassess the patient for prog-nostic stratification.

In this context, we propose a practical multi-step approach (Figure 3). Step 1 consists of early sepsis recognition through the integration of clinical presentation, biomarkers, and screening scores adapted to the geriatric population. Once sepsis is identified, Step 2 involves a comprehensive prognostic assessment to guide the level of care and define therapeutic goals; this should include frailty evaluation using validated tools such as the Frailty Index or the Clinical Frailty Scale (see prognostic assessment section). If active treatment is indicated, Step 3 emphasizes the prompt initiation of antibiotic therapy—within 1 h in septic shock and within 3 h in sepsis. Antibiotic therapy should be tailored according to the risk of multidrug-resistant organisms based on patient characteristics and local epidemiology, while ensuring appropriate dosing and minimizing drug toxicity. Step 4 involves evaluating the indication and feasibility of surgical or interventional source control, carefully balancing risks and benefits in light of frailty and comorbidities. Step 5 focuses on close monitoring of the clinical response to antimicrobial therapy, followed by Step 6, which entails early de-escalation based on microbiological findings. Finally, Step 7 highlights the importance of limiting antibiotic duration to the shortest effective course, promoting early switch to oral therapy when feasible and facilitating discharge and return to the home setting. Noteworthy, because this represents a dynamic clinical situation, it is important during the monitoring phase to reassess the patient for prognostic stratification, as indicated by the dashed line in Figure 3.

In particular, both research and clinical practice must advance along essential paths that effectively meet the particular needs of frail older individuals.

Frail older adults have been insufficiently represented in large-scale clinical studies, leading to their exclusion from the scientific evidence that guides treatment choices. Therefore, we need to encourage dedicated clinical trials, create guidelines that reflect their actual clinical characteristics, and establish antimicrobial stewardship initiatives specifically tailored for this group [10,144]. Based on these efforts, we can formulate recommendations that are genuinely aligned with the vulnerabilities, and care priorities of frail older adults.

Another important point is to invest in the creation of innovative and highly specific biomarkers to enhance diagnostic accuracy in the early detection of sepsis in frail older adults. For example, presepsin is a new biomarker that has demonstrated potential as a diagnostic and prognostic marker, even in older patients [145,146]. More reliable biomarkers would facilitate not only the timely identification of infections but also the accurate adjustment of antibiotic treatment duration, minimizing unnecessary drug exposure and the associated risks of resistance and toxicity.

Moreover, it is important to promote the development and validation of specific prognostic scores for frail older patients with sepsis. The existing scores often fail to fully capture the clinical complexity of these patients, where factors such as frailty, comorbidities, and functional reserve significantly influence outcomes. Dedicated prognostic tools would allow for a more rational allocation of resources, guiding decisions toward the most appropriate level of care and ensuring each patient receives proportionate and personalized care.

It is also fundamental to increase our comprehension of pharmacokinetic and pharmacodynamic (PK/PD) mechanisms in frail older individuals. This would enable us to optimize dosages, minimize toxicity, and maximize therapeutic outcomes in this special population [58].

Furthermore, the increasing prevalence of multidrug-resistant pathogens necessitates the creation of new molecules that possess enhanced efficacy and safety profiles, particularly for at-risk patients [46,147]. In this context, advancements in drug discovery, bolstered by machine learning [148] and artificial intelligence, are revealing exciting possibilities. The combination of clinical knowledge with cutting-edge technologies could drive the discovery of more effective and better-tolerated molecules, which would significantly enhance the management of infections in the vulnerable older population.

Finally, the adoption of machine learning is also becoming a promising advancement aimed at enhancing antibiotic prescribing practices and improving the effectiveness of antimicrobial stewardship [149]. This approach could be particularly beneficial for frail older adults, as machine learning models can concurrently integrate various clinical, laboratory, functional, and pharmacological factors—including frailty indices, renal and hepatic function, nutritional status, history of prior infections, and local resistance trends—to individually assess the likelihood of a bacterial infection, the threat posed by multidrug-resistant organisms, and the anticipated response to specific antibiotics. Furthermore, machine learning models are capable of suggesting dosages customized to the unique pharmacokinetic characteristics of each patient, estimating the risk of adverse effects, and guiding choices regarding the appropriate length of treatment to prevent unnecessarily extended or inappropriate antibiotic use.

## Figures and Tables

**Table 1:** Summary of observational studies investigating the performance of scoring systems for sepsis screening and prognostic assessment in older adults.

First Author	Year	Setting	Population	Scores Studied	Main Findings	Limitations
González Del Castillo [63]	2017	ED	Older (≥75 yrs) infected patients (*n* = 1071)	SIRS, qSOFA, GYM	GYM most sensitive; qSOFA specific but less sensitive	Selected population Limited generalizability
Bastoni [64]	2019	Acute geriatric unit	Older multimorbid patients (*n* = 272)	qSOFA, SIRS	Poor prognostic performance	Monocentric design Sample size
Ramos-Rincón [65]	2019	Acute care unit	Very old (≥80 yrs) bacteremia (*n* = 336)	qSOFA	qSOFA ≥ 2 predicts mortality	Retrospective design Selected population
Haas [62]	2020	ICU	Very old (≥80 years) sepsis patients (*n* = 5969)	qSOFA, SOFA, APACHE IV	qSOFA inferior to other ICU scores	Retrospective design Selected population
Boonmee [66]	2020	ED	Elderly sepsis patients (*n* = 1616)	SIRS, qSOFA, NEWS	qSOFA better but moderate prognostic accuracy	Retrospective and monocentric design
Falsetti [67]	2020	Acute care unit	Elderly with infection (*n* = 390)	SOFA, qSOFA	Low prognostic accuracy	Selected population
Remelli [68]	2021	Acute geriatric unit	Older sepsis patients (*n* = 165)	qSOFA, geriatric-qSOFA	Geriatric qSOFA better prognostic accuracy	Retrospective design Sample size Needs validation
Madrazo [69]	2021	Acute care unit	Older UTI patients (*n* = 282)	qSOFA, SOFA, SIRS	qSOFA better prognostic accuracy than SIRS, similar to SOFA	Selected population
Hernández-Quiles [70]	2022	Acute care unit	Very old (≥80 years) infected patients with (*n* = 336) and without (*n* = 336) bacteremia	qSOFA	Predicts long-term mortality	Retrospective design Case–control study
Brunetti [71]	2022	Acute Geriatric unit	Older infected patients (*n* = 230)	qSOFA, NEWS, MEWS	Comparable diagnostic accuracy. qSOFA useful to rule out sepsis	Monocentric design; sample size; definition of suspected infection (selection bias)
Brunetti [72]	2023	Acute Geriatric unit	Older infected patients (*n* = 305)	qSOFA, NEWS, MEWS	Comparable prognostic accuracy	Monocentric design; sample size; definition of suspected infection (selection bias)
García-Lamberechts [73]	2024	ED	Elderly patients (*n* = 6054)	qSOFA, NEWS, GYM	NEWS best predictor (qSOFA high specificity but low sensitivity)	Retrospective design Selected population
Okoye [24]	2025	Acute geriatric unit	Older sepsis patients (*n* = 93)	qSOFA	Incorporating frailty index and MDR status improves prediction	Monocentric design Sample size Selection bias

Footnotes: GYM score = Glasgow < 15; tachYpnea > 20 bpm; severe co-Morbidity as a Charlson index ≥ 3.

**Table 2:** Summary of clinical studies on frailty and adverse outcomes in patients with sepsis.

First Author	Year	Study Design	Setting	Population	Frailty Definition	Association with Clinical Outcomes
Fernando [15]	2019	Prospective study	ICU	Older adults with suspected infection (*n* = 1510)	CFS	Higher in-hospital mortality, longer ICU stay, increased risk of discharge to LT care, increased risk of readmission within 30 days, increased resource utilization and cost
Fernando [16]	2020	Prospective study	ED	Older adults (≥75 yrs) with suspected infection (*n* = 203)	CFS	Higher risk of septic shock and death within 30 days of ED admission
Haas [17]	2021	Multicenter prospective study	ICU	≥80 yrs with sepsis (*n* = 3596)	CFS	Higher 6-month mortality
Lee [18]	2022	Multicenter prospective study	ICU	Sepsis patients (*n* = 468 non-frail and 468 frail)	CFS	Higher in-hospital mortality
Patrizio [19]	2022	Prospective study	Acute geriatric unit	Older (≥70 yrs) sepsis patients (*n* = 240)	CGA, CFS, FI	Higher 6-month mortality
Dong [20]	2023	Prospective study	Acute geriatric unit	Older sepsis patients (*n* = 211)	CFS	Higher 1-year mortality, worse quality of life
Ding [21]	2024	Retrospective study	ICU	Septic shock patients (*n* = 9219)	FI-Lab	Higher in-hospital mortality
Li X [22]	2024	Retrospective study	ICU	Sepsis patients (*n* = 21,338)	mFI	Higher risk of adverse outcomes (delirium) and mortality
Zheng [23]	2025	Retrospective study	ICU	11,740 older adults	11-item mFI	Longer ICU stay and higher risk of delirium
Okoye [24]	2025	Prospective study	Acute geriatric unit	Older sepsis patients (*n* = 93)	CFS, PC-FI, 50-item FI	Higher mortality
Li Q [25]	2025	Cross-sectional study	ED	Older sepsis patients (*n* = 602)	CFS	Association with sarcopenia
Li D [26]	2025	Multicenter prospective study	ICU	Older sepsis patients (*n* = 1197)	FI-Lab	Higher 28-day mortality
Pellegrino [27]	2025	Retrospective study	Acute care unit	Patients with MDR Klebsiella bloodstream infection (*n* = 182)	FI-Lab	Higher risk of all-cause mortality, 28-day mortality and relapse
Ni [28]	2025	Systematic review & meta-analysis	Multiple settings	Sepsis/septic shock patients (*n* = 38.000)	Multiple frailty definitions	Longer ICU stay and non-significant trend toward increased mortality
Kutrani [29]	2026	Retrospective study	Hospital admission	Adult patients (*n* = 37,8916)	HFRS	Increased sepsis risk, longer length of stay and higher in-hospital mortality

## Data Availability

No new data were created or analyzed in this study. Data sharing is not applicable to this article.

## Abbreviations

ADRs, adverse drug reactions; AMS, antimicrobial stewardship; APACHE IV, Acute Physiology and Chronic Health Evaluation IV; CDI, *Clostridioides difficile* infection; CFS, Clinical Frailty Scale; CGA, Comprehensive Geriatric Assessment; COVID-19, coronavirus disease 2019; CR, carbapenem-resistant; CRP, C-reactive protein; ED, emergency department; ESBL, extended-spectrum β-lactamase; ESC, European Society of Cardiology; FI, Frailty Index; FI-Lab, Laboratory-based Frailty Index; GBD, Global Burden of Disease; HFRS, Hospital Frailty Risk Score; ICU, intensive care unit; LT care, long-term care; MDR, multidrug-resistant; MDROs, multidrug-resistant organisms; MEWS, Modified Early Warning Score; mFI, Modified Frailty Index; MRSA, methicillin-resistant *Staphylococcus aureus*; NEWS, National Early Warning Score; NEWS2, National Early Warning Score 2; PC-FI, Primary Care Frailty Index; PCR, polymerase chain reaction; PCT, procalcitonin; PK/PD, pharmacokinetic/pharmacodynamic; POET, Partial Oral Treatment of Endocarditis; qSOFA, quick Sequential Organ Failure Assessment; RCTs, randomized controlled trials; SIRS, Systemic Inflammatory Response Syndrome; SOFA, Sequential Organ Failure Assessment; TDM, therapeutic drug monitoring; UTI, urinary tract infection; VRE, vancomycin-resistant *Enterococcus* spp.
